# Sustainable strategies for Ebola virus disease outbreak preparedness in Africa: a case study on lessons learnt in countries neighbouring the Democratic Republic of the Congo

**DOI:** 10.1186/s40249-022-01040-5

**Published:** 2022-12-02

**Authors:** Caroline S. Ryan, Marie-Roseline D. Belizaire, Miriam Nanyunja, Olushayo Oluseun Olu, Yahaya Ali Ahmed, Anderson Latt, Matthew Tut Kol, Bertrand Bamuleke, Jayne Tusiime, Nadia Nsabimbona, Ishata Conteh, Shamiso Nyashanu, Patrick Otim Ramadan, Solomon Fisseha Woldetsadik, Jean-Pierre Mulunda Nkata, Jim T. Ntwari, Senya D. Nzeyimana, Leopold Ouedraogo, Georges Batona, Vedaste Ndahindwa, Elizabeth A. Mgamb, Magdalene Armah, Joseph Francis Wamala, Argata Guracha Guyo, Alex Yao Sokemawu Freeman, Alexander Chimbaru, Innocent Komakech, Muhau Kuku, Walter M. Firmino, Grace E. Saguti, Faraja Msemwa, Shikanga O-Tipo, Precious C. Kalubula, Ngoy Nsenga, Ambrose Otau Talisuna

**Affiliations:** 1 WHO Sub-Regional Office for Africa, Nairobi, Kenya; 2 WHO Country Office, Bangui, Central African Republic; 3 WHO Country Office, Juba, South Sudan; 4 WHO Regional Office for Africa, Brazzaville, Congo; 5 WHO Sub-Regional Office for Africa, Dakar, Senegal; 6 Africa Centres for Disease Control and Prevention, Addis Ababa, Ethiopia; 7 WHO Country Office, Brazzaville, Congo; 8 WHO Country Office, Bujumbura, Burundi; 9 WHO Country Office, Asmara, Eritrea; 10 WHO Country Office, Kigali, Rwanda; 11 WHO Country Office, Kampala, Uganda; 12 WHO Country Office, Luanda, Angola; 13 WHO Country Office, Dar Es Salaam, Tanzania; 14 WHO Country Office, Lusaka, Zambia

**Keywords:** Ebola virus disease, Emergency preparedness and response, Lessons learned, Case study, International Health Regulations, Democratic Republic of the Congo

## Abstract

**Background:**

From May 2018 to September 2022, the Democratic Republic of Congo (DRC) experienced seven Ebola virus disease (EVD) outbreaks within its borders. During the 10th EVD outbreak (2018–2020), the largest experienced in the DRC and the second largest and most prolonged EVD outbreak recorded globally, a WHO risk assessment identified nine countries bordering the DRC as moderate to high risk from cross border importation. These countries implemented varying levels of Ebola virus disease preparedness interventions. This case study highlights the gains and shortfalls with the Ebola virus disease preparedness interventions within the various contexts of these countries against the background of a renewed and growing commitment for global epidemic preparedness highlighted during recent World Health Assembly events.

**Main text:**

Several positive impacts from preparedness support to countries bordering the affected provinces in the DRC were identified, including development of sustained capacities which were leveraged upon to respond to the subsequent coronavirus disease 2019 (COVID-19) pandemic. Shortfalls such as lost opportunities for operationalizing cross-border regional preparedness collaboration and better integration of multidisciplinary perspectives, vertical approaches to response pillars such as surveillance, over dependence on external support and duplication of efforts especially in areas of capacity building were also identified. A recurrent theme that emerged from this case study is the propensity towards implementing short-term interventions during active Ebola virus disease outbreaks for preparedness rather than sustainable investment into strengthening systems for improved health security in alignment with IHR obligations, the Sustainable Development Goals and advocating global policy for addressing the larger structural determinants underscoring these outbreaks.

**Conclusions:**

Despite several international frameworks established at the global level for emergency preparedness, a shortfall exists between global policy and practice in countries at high risk of cross border transmission from persistent Ebola virus disease outbreaks in the Democratic Republic of Congo. With renewed global health commitment for country emergency preparedness resulting from the COVID-19 pandemic and cumulating in a resolution for a pandemic preparedness treaty, the time to review and address these gaps and provide recommendations for more sustainable and integrative approaches to emergency preparedness towards achieving global health security is now.

## Background

From May 2018 to September 2022, the Democratic Republic of Congo (DRC) experienced seven Ebola virus disease (EVD) outbreaks within its borders [1, 2]. Observations during the larger among these seven outbreaks showed that the country and most neighboring at-risk countries were not prepared despite lessons learnt from previous experience, not least the unprecedented West Africa EVD outbreak [3–5]. The revised International Health Regulations (IHR 2005) and several international policy frameworks have since been established to guide countries worldwide to build functional emergency preparedness and response capacities for all emergency events of significant threat to humans [6–13]^.^ However, developing the legal and regulatory mechanisms, physical infrastructure, human resources, tools and processes to meet IHR 2005 compliance assumes a foundation of a functional health system [14]. This is far from the reality in the context of Ebola endemic areas in the DRC and the majority of countries bordering these increasingly habitual events. The coronavirus disease 2019 (COVID-19) pandemic demonstrated to the world that policy guidelines for response capacities, even in high income countries with assumed robust health systems, did not necessarily translate into practice [15]. In view of the fact that countries in sub-Saharan Africa will continue to carry the burden of endemic and emerging infectious disease outbreaks including EVD outbreaks, it is imperative that lessons learnt from previous preparedness and response efforts to these events are well documented so as to inform future response efforts.

This case study thus highlights some of the gains and shortfalls with the EVD preparedness interventions against the background of the renewed and growing commitment for global epidemic preparedness highlighted during the 73^rd^, 74^th^ and 75^th^ World Health Assembly (WHA) events [16] and establishment of a new global hub for pandemic and epidemic intelligence [17]. Focusing specifically on countries neighbouring the DRC during that country’s tenth and eleventh EVD outbreaks, the findings of this case study were synthesized from various sources such as reports of joint monitoring exercises [18], simulations [19–21] and field support missions as well as descriptive reports captured during regular telecommunication sessions with country focal points and members of the field teams directly engaged with the coordination and roll out of EVD preparedness activities in priority countries. Many of the authors were also directly engaged in EVD preparedness activities and participated in coordination meetings at regional and country levels that provided insights into the key issues, challenges and lessons learnt from the EVD preparedness in the priority countries.

## The tenth and eleventh EVD outbreaks in the DRC (2018–2022)

The most recent outbreak of EVD in the DRC declared on August 21, 2022 is the fifteenth outbreak since the first EVD outbreak was reported in the country and globally in 1976 [2]. The ninth, eleventh and fourteenth outbreaks occurred in Equateur Province in Western DRC whilst the tenth, twelfth, thirteenth and fifteenth occurred within eastern DRC’s conflict affected Provinces of Ituri, North and South Kivu [22–26]. Among these, the tenth EVD outbreak was the world’s second largest and the first to occur within an active conflict zone, making it the most complex and prolonged experienced in the DRC [22]. The peak of the tenth outbreak occurred in April 2019 when up to 120 newly confirmed cases were reported weekly [22]. Over the 23-month period of the tenth outbreak, 29 health areas across 9 health zones in three provinces were affected, namely North and South Kivu and Ituri provinces. A total of 3463 cases including 2280 deaths, 1004 children (29%), 173 health workers, and 1171 recoveries were recorded [22].

The eleventh EVD outbreak was declared following laboratory confirmation of samples taken during investigation of a suspected cluster of deaths in Equateur Province on June 1, 2020 and prior to the ending of the tenth outbreak [26]. The nature of this outbreak was low intensity, but cases emerged sporadically across a broad area affecting 42 health areas in 13 of the 18 health zones of the province [21]. The wide geographical emergence of cases led to concerns of possible new introductions from zoonotic spillover or resurgence from viral persistence or latent infection from previously infected Ebola survivors as almost two thirds of confirmed cases were not registered contacts. From early October 2020 the number of reported cases reduced dramatically, and the outbreak was declared over on November 18, 2020. This outbreak recorded a total of 130 confirmed cases, 55 deaths and 75 recoveries [26].

## The high-risk countries neighbouring the DRC EVD outbreaks

Based on a World Health Organization risk assessment, nine countries sharing borders with the DRC during the tenth EVD outbreak were considered as moderate to high-risk and referred to as “priority countries”. The priority countries were further classified into priority one and two depending on their geographic proximity to the epi-center of the outbreak, volume of cross border movement and shared transport routes. As a result, Burundi, Rwanda, South Sudan and Uganda were identified as priority one countries and Angola, Central African Republic (CAR), Congo, Tanzania and Zambia as priority two.

Further consideration was given to the priority one countries based on their geographic, infrastructural, political and socio-economic contexts as determinants of their respective population vulnerabilities and health system capacities. Uganda had experienced six EVD outbreaks between 2000 and 2019 and demonstrated progressive capacity for detection and containment since the first and largest outbreak occurred in Northern Uganda in 2000 resulting in 224 deaths [27, 28]. This was evidenced during the DRC’s tenth EVD outbreak when three confirmed cases were rapidly identified and managed having crossed into Uganda in June 2019 [29]. South Sudan experienced three EVD outbreaks in 1976, 1979 and 2004 respectively within the former Sudan [30–32]. South Sudan and CAR are both conflict-affected countries experiencing protracted and complex humanitarian crises, internally displaced populations and highly fragile health systems. Communities in the priority countries are socially and economically interconnected and highly mobile across shared borders with the DRC and each other. Political stability in Uganda and Rwanda lend them to support hosting large numbers of refugees and access to cross border health and other public services.

Between October 2018 and December 2019, over 70 million USD was provided by the international donor community to the priority one countries for EVD preparedness [33]. Efforts to sustain preparedness capacities were retained up until the outbreak was declared over on June 25, 2020 despite dwindling resources. The priority countries updated their EVD National Contingency Plans in late 2019, some adopting a strategy for transitioning capacities developed during EVD preparedness to other public health emergencies. However, with the emergence of COVID-19 in early 2020, funding secured for implementation of 2020 transition plans was mostly repurposed to the COVID-19 response. The key pillars supported under EVD preparedness programmes in these countries were coordination, surveillance at the community, points of entry and health facility levels, laboratory diagnosis, case management, infection prevention and control, risk communications and community engagement, operational support and logistics and preventive vaccination of frontline health workers [34].

Using the same risk assessment criteria for allocating countries into priority one and two categories for EVD preparedness during the tenth EVD outbreak, the Republic of Congo and CAR were classified as priority one countries during DRC’s eleventh EVD outbreak. Despite their high-risk status and weak preparedness capacities, external funding to support either of these countries for EVD preparedness during the eleventh and fourteenth EVD outbreaks was negligible.

## Keys lessons from the EVD preparedness programmes in countries adjacent to the DRC outbreaks

The lessons learnt from the outbreaks captured the benefits of EVD preparedness in the countries bordering the DRC that were at high-risk of cross border transmission during the DRC’s tenth, eleventh and subsequent EVD outbreaks. The reports and experiences also captured several shortfalls in implementation processes. The gaps between building detection and response capacities in a sustainable manner in alignment with global policy frameworks as stipulated under Article 44 of the IHR 2005 and other international frameworks are highlighted and discussed.

## National and local capacity building during EVD outbreaks as a more sustainable approach to emergency preparedness

Experiences from the priority countries showed how national capacities built under several pillar areas were leveraged upon to respond to the COVID-19 response. Capacities in national and sub-national multi-sectoral coordination were strengthened, the need for institutionalization of infection prevention and control was realized and a heightened appreciation for the role of risk communication and community engagement in public health was acknowledged [28, 33, 34]. In South Sudan, surveillance capacities developed during EVD preparedness resulted in the early detection and aversion of a yellow fever outbreak in November 2018 [34]. Rwanda and Uganda reported that surveillance strengthened in the high-risk districts under EVD preparedness was easily translated to the COVID-19 response [35]. The extent to which the EVD preparedness investments mitigated the impact of subsequent outbreaks and the COVID-19 pandemic in the priority countries is an area requiring further exploration and quantification.

However, in some cases during these EVD outbreaks, some countries continue to depend on external intervention and a significant proportion of donor funding to conduct a response. For example, following emergence of the DRC’s eleventh EVD outbreak on June 1, 2020 in Equateur Province, little evidence of local capacity developed during the ninth outbreak, occurring in the same location less than two years earlier, was evident. Most of the response pillar areas had to be re-established and re-operationalized by external partners resulting in a delayed response. A similar situation was observed during the EVD outbreak in Guinea in 2021 and to a less extent in Beni in 2020 and 2021. One pillar area where the gap in national capacity is most evident is in critical care specific to managing EVD patients. In the majority of EVD outbreaks critical patient management was conducted by external partner organizations due to inadequately trained healthcare workers in critical care capacities within the health sector of sub-Saharan African countries [36].

Building local capacity from the existing pool of experienced human resources in countries and communities [36] or sharing this capacity between countries in the region was not fully exploited as a more sustainable investment in EVD preparedness at that time. While Rwanda sent a national team to Beni during the tenth EVD outbreak to acquire patient management capacity, CAR was unable to do this due to lack of funding during the DRCs eleventh outbreak. The gap in investment into building national critical care capacity became particularly evident during the COVID-19 response in several countries in the region [37, 38] when deployment of scarce international emergency medical teams was necessitated.

## Community participation and perceptions in EVD preparedness in the countries neighbouring DRC

During EVD preparedness interventions in the priority countries, the role of the local community was mostly limited to rumour tracking, community volunteer roles such as social mobilization activities, health promotion and community surveillance. There were lost opportunities to capture gender disaggregated data, community perspectives and suggestions on how existing local capacities and knowledge could be identified, leveraged upon and actively engaged in broader roles in preparedness and response operations. For example, communities in the DRC and some of the priority countries argued that large deployments of international personnel during EVD outbreaks undermined local expertise and little opportunity remained for integration and sustaining the skills developed during preparedness and response after these personnel left [39].

EVD preparedness interventions during the tenth outbreak benefitted from increasing integration between epidemiology, public health and clinical medicine with the social sciences (more specifically the discipline of anthropology) to understanding the social, cultural and political pathways of Ebola emergence and the perceptions of the community and individuals to the disease and their acceptance of the associated public health interventions. An increase in activities supporting and implementing participatory and evidence-based field work was observed in the neighbouring countries during the tenth EVD outbreak providing more nuanced understandings to inform more effective interventions [40, 41]. While anthropology is not a new discipline in Ebola response [42, 43], the utilization of anthropological findings to the extent of informing policies, programme planning and approaches to a broader array of interventions beyond communications and community engagement is yet to be fully realized as was evident from the preparedness activities in countries neighbouring the DRC.

## Quality of EVD preparedness and response capacity building interventions

EVD outbreaks are usually accompanied by rapid investment into the response phase allowing little time for formal planning of good quality training programmes. During the preparedness and response phase of these outbreaks, national staff and community volunteers were provided with basic trainings to implement field level activities under several pillar areas. Trainings were largely undertaken by an array of partner agencies in an ad hoc manner using multiple methods such as sensitizations, briefings, orientations, theoretical and practical workshops [28, 33]. An independent evaluation of EVD preparedness conducted in Uganda in 2020 identified how on occasions several partners were conducting trainings in the same area with the same theme and objectives [35]. While duplication is not a new phenomenon in development contexts, more effort is required for its identification and prevention through effective coordination mechanisms. In addition, mapping trainees, evaluating retention of their knowledge or application of the skills transferred in the medium to long term is seldom undertaken. Evidence that capacity is transferred into sustainable outcomes such as employment opportunities within the health system or engagement in other public health emergencies and improved quality of care beyond periods of active EVD response are also lacking.

There were few examples demonstrating effective coordination of EVD preparedness trainings or establishment of formal procedures for reviewing the structure, mode of delivery and quality of the training content, assessing the capacity of the trainers or selection process of the participants. The collaboration between the academic and public health sectors for EVD preparedness capacity in the priority countries during the tenth EVD outbreak was a new development and represented an opportunity for countries to encourage inter-regional partnerships between higher level institutions to ensure institutionalization of training programmes for EVD outbreak preparedness and response.

## Increased recognition of the need for sustainable context appropriate research and innovations

The importance of research and development in EVD outbreaks cannot be overemphasized, particularly the development of vaccines and therapies for high impact pathogens including EVD [44]. The low intensity outbreaks observed during DRC’s eleventh, twelfth, thirteenth and fourteenth EVD outbreaks may have resulted from the roll out of the investigative rVSV-ZEBOV-GP Zaire ebolavirus vaccine during the ninth and tenth outbreaks in these same locations. Studies on the duration of vaccine efficacy are ongoing [45–47]. However, a key concerning observation during these outbreaks, is that while funding to support research and development of experimental therapies and vaccines during EVD outbreaks is high, support to these same communities to access these life-saving and oftentimes unaffordable products after licensing is limited.

Observations show that countries seeking vaccination of high-risk health workers in districts bordering the eleventh, twelfth and thirteenth outbreaks failed to access adequate quantities of the licensed vaccine. Furthermore, little attention was targeted towards local clinical research into homegrown interventions which could reduce mortality in low resource settings. For example, allocation of research funding to explore the contribution of supportive care interventions such as blood volume enhancers including intravenous fluids, colloid and crystalloid solutions, blood transfusion and parenteral nutrition as life-saving interventions in patient management during EVD preparedness was lacking in the DRC and other at-risk countries during the EVD outbreaks.

EVD outbreaks were frequently accompanied by a cascade of medico-technical innovations such as the use of robots to monitor patient body temperatures at airports in some African countries and piloting drones to transport EVD alert samples across the equatorial forests of Equateur Province raising serious safety concerns are some examples. Whilst technological interventions can address gaps in response operations and reduce costs, such innovations need to be informed by public health principles.

## Sustainable solutions for EVD case management

When the eleventh EVD outbreak emerged in Equateur Province at the end of May 2020, temporary facilities constructed during the ninth outbreak to isolate and treat patients either no longer existed or were unfit for purpose. This demanded rapid construction of inferior quality structures that were poorly managed in the initial phase of the response due to weak partner co-ordination. In some communities, in addition to fear and stigma, reports of poor patient care of those admitted at the treatment centers translated into additional fear, avoidance to report alerts and resistance to allow access to safe and dignified burial teams to respond to community deaths. Reversing mistrust required considerable time and investment, not only by improving the physical infrastructure and system of care but through intensive community engagement. In the 2021 EVD outbreak in Guinea mistrust remained among communities from bad experiences associated with Ebola Treatment Centers (ETCs) during the West African outbreak (2014–2016) [48].

The design and materials used to construct ETCs have witnessed several innovative improvements in the past decade. These innovations materialized in response to the need for improved health worker and patient safety, improved comfort and visibility for the patient of their surroundings and for family members, reduced time constraints to monitor and treat patients and a reduction in personal protective equipment (PPE) use and waste management costs without compromising infection prevention. The concept of transit centers introduced in North Kivu during the tenth EVD outbreak using semi-permanent materials as extensions to existing permanent structures helped to buffer community fear.

Building permanent or semi-permanent structures for isolating and treating patients versus temporary structures became an area of considerable debate between donors and countries throughout EVD preparedness efforts in the at-risk countries. The argument for not investing in infrastructural projects lies in the assumption that such projects are highly costly, take time to complete and funds might be misused or diluted into a larger funding pool resulting in a poor-quality product that does not serve the intended purpose. Another argument against permanent structures is that the location of an EVD outbreak cannot be known in advance, therefore constructing permanent buildings in several locations in expectation of a confirmed case is not feasible. Temporary structures are considered suitable to serve the purpose for the duration of the project and pose a lower risk for the investment. However, in practice it has been shown that the cost of constructing and maintaining a temporary ETC is equivalent and (in some cases) more costly to construct and maintain than semi-permanent/permanent structures. Temporary structures inevitably decompose over time due to exposure to weather and represent poor value for money. Many of the at-risk countries expressed preference for semi-permanent/permanent structures or renovation of existing buildings as a more sustainable and cost-effective solution that can be repurposed to general isolation facilities for other infectious diseases following EVD outbreaks. In South Sudan, the semi-permanent structure built during EVD preparedness in 2019 was sustained and continued to be used throughout the COVID-19 response [32]; this design was adopted for use in other countries such as Burundi.

However, despite attempts to humanize ETC design in recent years, all EVD outbreaks in the DRC between 2018 and 2022 continue to see patients treated in temporary structures. The consensus that emerged is that there is “no one size fits all solution”. Each outbreak evolves and behaves differently and can emerge in a variety of contexts warranting different design options. Decision making processes at country level requires further dialogue and review of case studies from EVD preparedness inclusive of community perspectives.

## Integrating EVD readiness into existing health systems and programmes

The large influx of funding and resources associated with epidemics has in the past resulted in the duplication of efforts and implementation of short-term interventions that fail to strengthen country capacity in the longer term often representing missed opportunities. A key lesson from the EVD preparedness in the priority countries was the need to leverage existing health programmes and identify existing systems and use them as entry points for integrating EVD preparedness. This is more pertinent in view of the more recent discovery that latent EVD infections lasting for several years could trigger outbreaks as was seen in the case of the 2020–2021 outbreaks in Guinea [50, 51]. This further underscores the need for longer-term and sustainable approaches to EVD preparedness in Africa.

Another example was the need for enhanced integration of EVD surveillance into the Integrated Disease Surveillance and Response (IDSR) system, the primary disease surveillance system for the general population, used in the priority countries. In South Sudan for example, a project approach under EVD preparedness resulted in an EVD alert management system limited to high-risk districts for EVD that ran parallel to the existing IDSR system [33]. This was acknowledged as a lost opportunity that could have integrated EVD surveillance into nationwide training of health workers and roll out of the third Edition IDSR for all priority infectious diseases to health facilities in the country. Although the detection and aversion of a yellow fever outbreak identified under EVD surveillance was a benefit of the vertical surveillance model described above, the system was not sustained beyond the funding period.

A similar observation was noted for enhancing community surveillance which gained a lot of traction during EVD preparedness, but its momentum waned following withdrawal of Ebola specific funding [28]. This is largely due to funding reporting mechanisms which demand results on the performance of specific activities under vertical disease programmes within a fixed time frame dictated by the length of the funding period. This approach encourages “new” or duplicated systems that undermine existing systems and resources, particularly the role of the community, that could be leveraged upon. Such lost opportunities justify a need for more coordinated, informed and negotiated planning processes.

## Impact of conflict on EVD preparedness in the countries neighbouring DRC

South Sudan and CAR are both conflict-affected countries experiencing protracted and complex humanitarian crises, internally displaced populations and highly fragile health systems. Uganda, Rwanda and Burundi host large numbers of refugees. This presence of internally displaced persons and refugees in the countries undergoing preparedness presented special challenges [33]. First, implementing preventive interventions such as hand washing was constrained by limited access to water and sanitation facilities in these populations. Second, crowded living conditions made social distancing and isolation of sick family members impracticable in such settings. Third, the weak health services in the displaced population in particular in the conflict affected countries in general constrained effective surveillance, infection prevention and control and other preparedness intervention. Fourth, the insecurity restricted free movement of staff and supplies in the high-risk areas thus limiting the geographic scope of the preparedness interventions in some cases. Nevertheless, South Sudan reported being able to overcome some of these challenges through available opportunities and the use of innovative approaches [33, 34].

## Cross border collaboration and regional coordination of EVD preparedness efforts

Cross border collaboration and regional coordination of preparedness and response efforts for EVD outbreaks is a critical factor in containing cross border transmission of outbreaks. Observations showed that progress in this preparedness area varied across the neighbouring countries during the tenth and eleventh EVD outbreaks in the DRC. In Uganda and Rwanda, the Ministries of Health successfully organized cross border meetings and signed Memoranda of Understanding for cross border collaboration with their counterparts in the DRC [28] which facilitated cross border surveillance, sharing of information and timely detection of cross border transmission of infection [29, 35]. However, this was not the case in South Sudan largely due to ongoing conflicts and insecurity around the South Sudan/DRC border and the huge distance between the national and sub-national coordination hubs of the two countries. This was a missed opportunity for synchronization of preparedness interventions particularly surveillance, case management and immunization.

Although several regional coordination meetings involving the DRC and the priority countries were held [49], these were one-off events where plans were initiated but did not translate into ongoing regional coordination of preparedness efforts. A partners’ regional coordination platform based in Nairobi, Kenya was established but towards the end of the tenth EVD outbreak in October 2019 when momentum for EVD preparedness investment was waning. Political will for cross-border and inter-regional coordination efforts was high on government agendas at this point and donors were willing to support ongoing activities to facilitate lessons learned symposiums, cross border simulation exercises and after action reviews. Unfortunately this critical period for reflection on EVD preparedness following the largest and most complex of the DRC outbreaks that posed the highest risk to the sub-region was rapidly overshadowed by the emergence of the COVID-19 response in early 2020.

## Reflections on the lessons learned

A recurrent theme that emerged in the lessons learnt from EVD preparedness in countries bordering the DRC EVD outbreaks is a propensity towards implementing short-term vertical interventions during EVD outbreak preparedness and response rather than sustainable investment into strengthening systems for health security in alignment with IHR 2005 obligations, Universal Health Coverage and the Sustainable Development Goals (SDGs).

Since the first recorded EVD outbreaks in 1976, response interventions have been mostly reliant on international emergency funding and expertise. As a result, resources are limited to the timeframe of the outbreak period and withdrawn once the outbreak is declared over. In contexts such as the DRC and its neighbouring countries known to frequently experience or be at high-risk of EVD outbreaks, negligible national investments have been made to establish foundational preparedness elements on which emergency responses can rapidly become operational prior to arrival of external support. Unfortunately, the current approach for supporting EVD preparedness, follow a declared outbreak and is an extension of the response. Also, limiting EVD preparedness support to “operational readiness” after emergence of a nearby outbreak risks undermining the importance and volume of work required to build the foundation of preparedness in countries, particularly in contexts where weak health systems exist. Even more concerning than limiting EVD preparedness support is absence of support for preparedness in high-risk areas bordering outbreaks as observed in relation to the majority of DRC outbreaks following the tenth EVD outbreak. This is particularly concerning for CAR and Congo bordering Equatorial Province in western DRC, where no significant investment towards implementing EVD preparedness has occurred despite experiencing three outbreaks since 2018 across a shared landscape.

While extending EVD response capacities to support preparedness appears to make sense at several levels it has several limitations. In support of the argument, preparedness benefits from being an extension of a response in that having expertise, experience and capacities available during the response can inform and guide inputs and activities required for coordinating preparedness activities simultaneously. Also, the proximity of a response influences increased alert reporting in neighbouring areas due to enhanced awareness and surveillance activities. This also increases willingness of neighbouring countries to engage in preparedness activities and cross-border collaboration and finally it highlights gaps and funding needs. However, when EVD preparedness is limited to being an extension of the response, it falls within the timeline allocated for supporting the response. This does not allow sufficient time to develop national capacities and skills for countries to affect an independent response or generate a baseline of resilience to mitigate future events. Once the outbreak is declared over, support for the response and preparedness efforts initiated in bordering countries is withdrawn leaving these health systems in much the same state as prior to the outbreak. As the DRC’s tenth EVD outbreak phased down in the latter part of 2019, funding for additional EVD contingency plans waned, yet several gaps remained for the priority countries to reach a minimum level of capacity. Another limitation is that when preparedness support is an extension of a response it can become defined by the response, resulting in carbon copied approaches and activities, some of which tend to be reactive in nature allowing little scope to conceptualize more sustainable or context appropriate methods.

Constructing temporary ETCs versus permanent structures in EVD prone contexts is one example highlighted above. If issues such as ETC design, incentive payments to responders, mapping and co-ordination of local capacities, capturing community insight on their understanding and application of EVD preparedness measures and vaccination strategies were explored and resolved at country level outside the urgency of a response environment, analysis of previous case studies and lessons learnt could be more effectively reviewed to inform decision-making and planning processes for future responses.

In the event of public health threats with capacity to cross borders, building core capacities to prevent, detect and implement a public health response is a mandatory requirement of Member States under the revised IHR. However, the renewed commitment for post COVID-19 pandemic preparedness needs to look beyond the confines of a global health security agenda. Other factors such as the social determinants of health, local culture and human behavior are also critical in this regard. Poverty and its associated challenges of poor living conditions, inadequate access to water and sanitation, illiteracy, engagement in alternative means of sourcing food and livelihood such as forest activities and inaccessibility to conventional health services is one example why the poor are increasingly vulnerable to increasing EVD incidence in endemic areas. The global community including governments, regional blocks and development agencies thus need to look beyond the health aspects of EVD outbreaks and focus on the linkages between the social and political determinants of high impact disease emergence.

Some recommendations to inform more sustainable preparedness approaches for future EVD preparedness investments into EVD and other viral hemorrhagic fever (VHF) disease prone contexts have emerged from this case study are outline in Box 1.

**Box 1:** Recommendations

• Enhancing interdisciplinary approaches between epidemiology, public health, clinical medicine and the social sciences to generate more nuanced understandings of EVD emergence and the heterogeneity that exists among community perceptions can inform more appropriate preparedness and response interventions. • Mapping of local capacities, inclusion of community perspectives and anthropological methods in preparedness, resource allocation and operationalization of EVD and other infectious disease outbreaks is important. • National accreditation and quality assurance of training content for emergency responders, post training evaluation and registration of trainees in communities is imperative. Capacity and funding should be increased for local research and development into emerging areas of EVD such as affordable treatment innovations, detection of new virus strains and better understanding of the transmission dynamics during future outbreaks. Accompanying this should be advocacy to ensure the most vulnerable populations have priority access to vaccines and new technologies. • Permanent/semi-permanent infrastructural development for infectious disease isolation and treatment units near existing health facilities in EVD prone communities should be encouraged. • EVD preparedness pillars should be integrated into routine health programmes as much as practical. • Investments into the development and integration of human and veterinary public health surveillance and laboratory services for early detection and identifying new virus type variants should be encouraged within the One Health framework. • Investments into survivor care programmes to support Ebola survivors and monitor potential virus persistence or latent infection and transmission events should continue. • Greater support and funding should be provided for cross border and inter-regional coordination, collaborative retrospective reviews and cross border collaboration on EVD and other high impact disease outbreaks.	

## Conclusions

Global health security and health system strengthening are two sides of the same coin. Unfortunately, the lessons learnt from this case study demonstrate that rapid and temporary preparedness and mitigation measures in reaction to EVD outbreaks threatening international borders and regional health security on the Africa continent continue to be the preferred approach to EVD preparedness in high-risk contexts. Hard lessons learnt including those highlighted in this paper should drive advocacy for a shift from reactionary and short-lived interventions towards more sustainable long-term approaches to EVD and emergency preparedness which would build health system resilience in general. A starting point would be for countries including representatives from previously affected communities and global actors to create a space where the much-needed open dialogue can occur to review these and other best practices and lessons learnt around EVD preparedness long before outbreaks occur. From here resolutions, contextual view-points and recommendations can evolve to disentangle recurrent bottlenecks that emerge time and again during EVD response and preparedness. The time for this open dialogue, engagement and inclusive collaboration is now.

## Data Availability

Not applicable.

## Abbreviations

CAR: Central African Republic
COVID-19: Coronavirus disease
DRC: Democratic Republic of the Congo
ETC: Ebola Treatment Centre
EVD: Ebola Virus Disease
IDSR: Integrated Disease Surveillance and Response system
IHR (2005): International Health Regulations (2005)
MoH: Ministry of Health
PPE: Personal Protective Equipment
SDGs: Sustainable Development Goals
WHA: World Health Assembly
WHO: World Health Organization
VHF: Viral Hemorrhagic Fevers
